# Addressable Acoustic Actuation of 3D Printed Soft Robotic Microsystems

**DOI:** 10.1002/advs.202001120

**Published:** 2020-09-21

**Authors:** Murat Kaynak, Pietro Dirix, Mahmut Selman Sakar

**Affiliations:** ^1^ Institute of Mechanical Engineering Ecole Polytechnique Fédérale de Lausanne Lausanne CH‐1015 Switzerland

**Keywords:** acoustic waves, biomanipulation, direct laser writing, mechanical design, soft robotics

## Abstract

A design, manufacturing, and control methodology is presented for the transduction of ultrasound into frequency‐selective actuation of multibody hydrogel mechanical systems. The modular design of compliant mechanisms is compatible with direct laser writing and the multiple degrees of freedom actuation scheme does not require incorporation of any specific material such as air bubbles. These features pave the way for the development of active scaffolds and soft robotic microsystems from biomaterials with tailored performance and functionality. Finite element analysis and computational fluid dynamics are used to quantitatively predict the performance of acoustically powered hydrogels immersed in fluid and guide the design process. The outcome is the remotely controlled operation of a repertoire of untethered biomanipulation tools including monolithic compound micromachinery with multiple pumps connected to various functional devices. The potential of the presented technology for minimally invasive diagnosis and targeted therapy is demonstrated by a soft microrobot that can on‐demand collect, encapsulate, and process microscopic samples.

## Graphical abstract

A soft microrobot construction kit is introduced, which constitutes untethered transducers such as pumps and rotors and a variety of machine parts. The compound micromachinery is printed as a single piece from a biocompatible hydrogel using two‐photon polymerization and actuated wirelessly using acoustic waves. Frequency‐selective acoustic excitation of several transducers on the same robot enables dexterous biomanipulation.

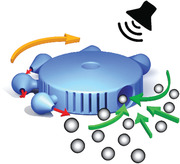

Microfabricated devices have led to revolutionary changes in our ability to manipulate small volumes of fluid and microscopic samples contained therein.^[^ ^1^ ^]^ As a result, majority of state‐of‐the‐art in vitro biomedical platforms contain microfluidic components. Operating these devices requires the use of bulky pumps, compressors, or tethered electrical powering units, which significantly increase the overall size and limit the portability. A key technological challenge has been the development of untethered microfluidic systems that are capable of providing such functionality with wireless control for in vivo applications. Ideally, such systems are expected to determine the timing, duration, and dosage of the intervention and allow remote, noninvasive, repeatable, and reliable control of diagnostic or therapeutic procedures.^[^ ^2^, ^3^, ^4^, ^5^, ^6^, ^7^, ^8^ ^]^ Remote control has been recently achieved with flexible piezoelectric actuators powered by tiny batteries or magnetic induction.^[^ ^9^, ^10^, ^11^, ^12^ ^]^ However, the sizes of these electromechanical devices are still in the centimeter range. One potential strategy to address the miniaturization challenge is building automata that operate only with mechanical components. While direct miniaturization of the clockwork mechanisms that power macroscale automata is not a viable option, flexible structures fabricated from natural or synthetic hydrogels possess physical intelligence in the absence of electronic components.^[^ ^13^, ^14^, ^15^, ^16^, ^17^ ^]^

There exists an engine at the heart of every modern machine that is connected to various mechanisms for the controlled application of forces and creation of motion. Development of remotely powered microscopic engines from hydrogels will resolve the aforementioned bottleneck and establish the sensing and actuation capabilities of lab‐on‐a‐chip technology on board an implantable compartmentalized soft microrobot. Two‐photon polymerization emerged as a feasible solution for printing polymers in complex forms with nanometer scale resolution.^[^ ^18^, ^19^, ^20^ ^]^ While 3D printed, magnetically driven microscopic screws and gears provide an effective method for mass transport at low Reynolds number,^[^ ^21^, ^22^, ^23^ ^]^ selective magnetization and operation of moving parts require complex manufacturing steps. Furthermore, magnetization scales with volume, thus, microscale structures fabricated from magnetic nanocomposites or polymers coated with magnetic thin films generate very limited thrust and fluid flow. As an alternative strategy, laser power has been harnessed to actuate 3D printed microstructures.^[^ ^24^, ^25^, ^26^, ^27^ ^]^ However, focusing the laser beam continuously and precisely at a small region through tissues and organs without causing excessive heating impedes further development.

Bubbles and sharp‐edged solid structures excited by acoustic waves generate steady streaming in liquids,^[^ ^28^, ^29^, ^30^, ^31^ ^]^ providing a minimally invasive and scalable solution for powering untethered micromachines in vivo.^[^ ^32^, ^33^, ^34^, ^35^, ^36^ ^]^ While bubbles are quite efficient in transducing acoustic energy,^[^ ^37^ ^]^ microrobotic systems actuated by entrapped bubbles work reliable only for hours.^[^ ^34^, ^35^ ^]^ The size and mechanical response of bubbles do not stay the same under physiological conditions, thereby gradually shifting the resonance frequency of the actuators and deteriorating the performance of the machine. Furthermore, stable entrapment and precise actuation of multiple bubbles inside a compartmentalized microrobot is quite challenging due to surface effects. On the other hand, actuation based on specially designed structures provides a durable and versatile solution that delivers the same performance over time and under varying environmental conditions. Previous work demonstrated the feasibility of shaping hydrogels into solid triangular beams using projection lithography inside microfluidic channels and actuating them to drive untethered swimmers and rotors using acoustic waves.^[^ ^36^, ^38^ ^]^ However, this fabrication technique does not allow development of morphologically complex dexterous microrobots, restricting its use to on‐chip planar manipulation.

Here, we introduce an integrated design and fabrication methodology for the spatiotemporally resolved, frequency addressable acoustic actuation of 3D hydrogel microrobots. Once excited periodically at its resonance frequency, a microstructure with sharp features submerged in fluid generates a pair of counter‐rotating vortices and a localized jet as a manifestation of viscous streaming.^[^ ^39^ ^]^ This principle constitutes the foundation of our actuation methodology. Inspired by the form and ease of operation of macroscale pump‐jets, we developed a microjet (µjet) engine that transports fluid through local body deformation without moving parts. The µjet engine consists of a cylindrical casing surrounding a conical flexible wedge that was obtained by the 360° rotation of an inclined sharp‐edged beam (**Figure** **1**a). This axisymmetric configuration ensured zero average torque on the main body during operation while concentrating the synthetic jet along the longitudinal axis. Furthermore, this design scheme allowed all the machine components to be printed as a single piece from a biocompatible hydrogel, poly(ethylene glycol)diacrylate (PEGDA), using two‐photon polymerization (Figure 1b).

**Figure 1:**
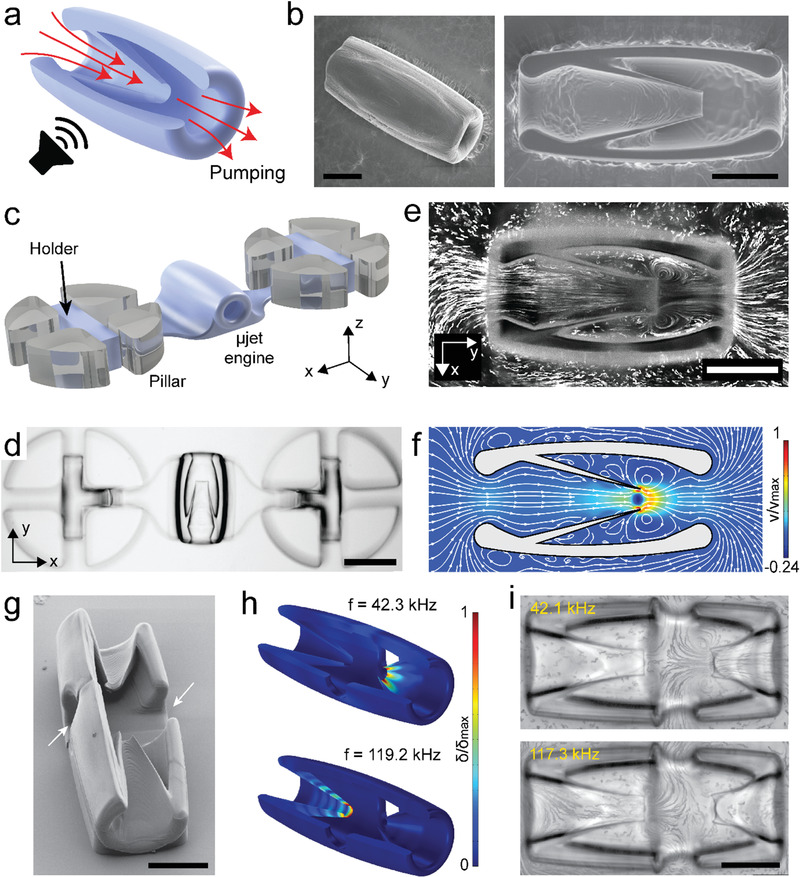
The fabrication and operation of acoustically excited µjet engines. a) Schematic representation of the working principle of the µjet engine. The red arrows show the direction of pumping generated by acoustic streaming inside the device. b) Electron microscopy images showing fully (left) and partially (right) printed µjet engines. c) Illustration of the microfluidic test platform. The holder was printed along with the µjet engine to stabilize the motion. d) Representative bright‐field image of a 3D‐printed µjet engine that is anchored to pillars. e,f) Streamlines inside and around the µjet engine are visualized experimentally using fluorescent microparticles (e) and numerically using CFD simulations (f), respectively. The localized microstreaming around the tip of the conical wedge results in jet in the middle of the device. g) Electron microscopy image of the bidirectional µjet engine. The arrows show the outlets of the pump. h) Numerical simulations show addressable acoustic excitation of the µjet engine within the same device at different frequencies. Figure S5 in the Supporting Information shows corresponding images with exaggerated deformation. i) Streamlines showing the flow generated at the resonance frequency of each µjet engine. Scale bars: 75 µm in (b), (e), and (i) and 150 µm in (d).

Quantitative analysis of the flow generated around the engine is instrumental for the optimization of the pumping performance. This procedure requires robust immobilization of the µjet engine inside a closed test chamber. We fabricated a microfluidic chamber with elastomer pillars that served as ports for the docking of µjet engine (Figure 1c). Cross‐shaped cavities engraved inside the pillars and T‐shaped hydrogel holders printed on both sides of the engine lock the system in place. This configuration resembles the facilities used for testing macroscale jet engines where the engine is mounted on a thrust frame to prevent forward motion. The length of the holder arms was kept long enough to have negligible hydrodynamic interactions between the pillars and the µjet engine. The holders had a nonuniform profile with a thickness starting from 8 µm outside the docking site until reaching the pillars to minimally distort the shape of the engine. We left a minimum clearance of 50 µm between the top of the chassis and the ceiling of the microchannel to avoid formation of underpolymerized sections due to the oxide layer. Parameter sweeps for the laser power and scanning speed were undertaken for successful printing of fine features. At low laser power and high scan speed, the prepolymer solution was not uniformly polymerized everywhere, resulting in differential swelling of the hydrogel. This inhomogeneity‐induced residual stress and bending of structures, specifically at the sharp tip. On the other hand, high laser power and low scan speed resulted in overpolymerization, which occasionally led to clogging of the pump. Figure 1d shows a representative bright‐field image of an engine immobilized inside the microfluidic testing platform.

We injected 1 µm polystyrene particles into the channel and recorded flow upon acoustic excitation at high frame rates (Movie S1, Supporting Information). Streamlines were visualized by rendering temporal projection of images from time‐lapse movies (Figure 1e). Instantaneous flow velocity was extracted from the particle movement in the middle plane of the engine ≈30–50 µm behind the sharp tip, a region we denote as the observation site (Figure S1a, Supporting Information). The flow velocity was tuned by adjusting the intensity of the applied acoustic field, i.e., the voltage applied to the piezoelectric transducer. The flow along the longitudinal axis of the pump reached velocities as high as 476 ± 71 µm s^−1^ at 116 kHz at a peak‐to‐peak input voltage (*V*_PP_) of 55 V in the observation site. As a manifestation of low Reynolds number, the pumping completely began and halted within milliseconds after turning on and off the input source, respectively. The amplitude of the structural oscillations was not large enough to generate pumping below *V*_PP_ = 2.5 V. Above *V*_PP_ = 2.5 V, the pumping velocity at the observation site displayed a quadratic relationship with the input voltage (Figure S1b, Supporting Information). A linear relationship exists between *V*_PP_ applied to the transducer and the generated acoustic pressure. The acoustic energy density in the workspace quadratically increases with increasing pressure^[^ ^40^, ^41^ ^]^ while the streaming velocity generated by an oscillating sharp‐edged structure changes linearly with the acoustic power.^[^ ^31^ ^]^ To explore acoustic streaming around the flexible wedge, we recorded movies of particles at different focal planes. Streamlines showed steady streaming all around the vibrating tip of the wedge, essentially forming a vortex ring (Movie S2 and Figure S2, Supporting Information).

We next performed a numerical modal analysis of the µjet engine and associated fluid flows using finite element analysis (FEA) and computational fluid dynamics (CFD), respectively. The analysis was performed on the 3D machines where the influence of the fluid was incorporated to the model through added mass. At certain natural frequencies, the corresponding vibration modes showed large displacement at the tip of the wedge while reporting negligible deformation on the rest of the structure (Figure S3, Supporting Information). This particular deformation profile is expected to be optimal for thrust generation, and, thus, we operated the engines around the corresponding frequencies. Solving the Navier–Stokes equations in 3D requires significant computational power, particularly when considering deformable mesh and moving boundaries. Only axisymmetric modes were considered so that the simulations could be performed on 2D meshes and, as a result, the computational cost of the CFD simulations would be reduced significantly. The simulation framework consisted of a one‐way fluid‐structure interaction model where the stresses on the structure from the fluid were not considered. For a given eigenfrequency, the eigenmode was extracted from the FEA and applied as a time‐dependent displacement condition on the structure boundary (i.e., moving boundary) in the CFD. We tuned the amplitude of deformation and simulated the flow patterns around the oscillating structures. Simulation results captured counter‐rotating vortices around the flexible wedge (Figure 1f) and the flow profile was consistent with experimental data shown in Figure 1e. We tuned the displacement of the tip in the computational model to match the empirical fluid flow recorded at 116 kHz, which gave an order of magnitude estimate for the amplitude of structural vibrations. At *V*_PP_ = 55 V, the empirical value of the flow velocity was 500 µm s^−1^ which corresponds to 0.15 µm tip displacement in the computation model (Figure S4, Supporting Information).

The ability to regulate mechanical power by tuning the amplitude and frequency of the traveling acoustic waves enables the operation of microfluidic systems with multiple engines. We designed two engines with different operation frequencies (i.e., pumping generating eigenfrequencies) by changing the length of the wedge while keeping the opening constant (Figure 1g). Both engines were printed together on the same casing with a common outlet. This configuration allowed formation of a cross junction where the profile of the output flow depended on the frequency of the acoustic wave. Although engines were physically attached to each other, the modal analysis showed that it was feasible to selectively operate engines at different frequencies (Figure 1h). Streamlines recorded at resonance frequencies corresponding to aforementioned vibration modes are shown in Figure 1i. Flow velocities recorded at the exhaust of the pumps were *v*_1_ = 7.64 ± 1 µm s^−1^ and *v*_2_ = 58.6 ± 3.3 µm s^−1^ at 42.1 kHz while *v*_1_ = 37.75 ± 3.37 µm s^−1^ and *v*_2_ = 0 µm s^−1^ at 117.3 kHz. This frequency dependent transition in flow direction led to an almost digital switch between the two inlet ports (Movie S3, Supporting Information).

Microfluidic manipulation systems require not only pumps but also passive and active control elements such as valves and mixers that are seamlessly integrated on the same platform. Expelling mass could provide effective means for the actuation of hydraulic control elements. Macroscale thrusters are propulsive devices used by watercraft for very rapid and accurate control of vessels. They also provide impressive maneuverability with compact and lightweight gear. Inspired by these machines, we engineered a microscopic thruster (µthruster) in the form of an empty hydrogel container with a single opening (**Figure** **2**a). At certain frequencies, the graded structure concentrates the deformation at the opening of the machine much like the flexible wedge of the µjet engine (Figure 2b). The jet emanating produces thrust in accordance with the law of conservation of momentum. The streamlines visualized the collection of fluid from the periphery and localized expulsion along the centerline (Figure 2c). The rationale behind the formation of a cavity was to reduce the amount of material necessary for printing and increase the amplitude of oscillations at the tip.

**Figure 2:**
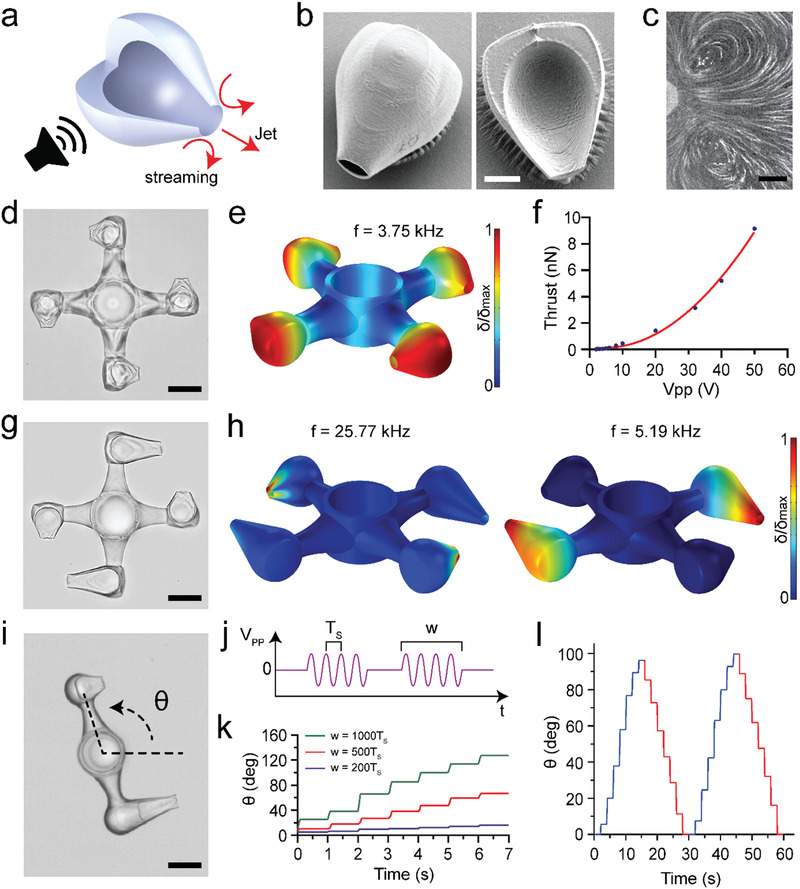
The fabrication and operation of µthrusters. a) Schematic representation of the working principle. The red arrows show the direction of acoustic streaming and jet flow. b) Electron microscopy images of a fully (left) and partially (right) printed devices. c) Streamlines showing counter‐rotating vortices at the tip of a µthruster mounted inside the test chamber. d) Bright‐field image of a µrotor propelled by four µthrusters. The device was printed in situ around an elastomer pillar. e) Numerical simulation of the µrotor showing the eigenmode corresponding to the acoustic excitation frequency used in the experiments. Figure S6 in the Supporting Information shows corresponding images with exaggerated deformation. f) The calculated thrust of a µthruster with respect to input voltage used in the experiments. g) Bright‐field image of a bidirectional µrotor driven by two different designs of µthruster. The symmetric arrangement ensures smooth rotation. h) Numerical simulations showing selective excitation of µthrusters at different resonance frequencies. Figure S6 in the Supporting Information shows corresponding images with exaggerated deformation. i) Precise angular position control using pulse‐width modulation. j) The input signal consists of pulsed sine waves with period *T*_S_ and frequency *f*_S_. The pulse width (*w*) was modulated while the amplitude and period of the pulse were kept constant. k) CCW rotation of the two‐arm µrotor exited with pulses of varying *w*. l) Bidirectional rotation of the device with pulse‐width modulation. CW rotation was performed at *f*_S_ = 4.6 kHz and with *w* = 1000 *T*_s_ while, for CCW rotation, the input signal was tuned to *f*_S_ = 23.8 kHz and *w* = 1200 *T*_s_ to generate identical motion in both directions. Scale bars: 15 µm in (b), 25 µm in (c), 50 µm in (d), (g), and (i).

Compared to the µjet engine design, µthruster has a significantly smaller footprint, enabling the development of compound micromachines with multiple actuators. We developed a microscale rotor (µrotor) driven by several µthrusters that were arranged around the arms in a way to increase the total torque (Movie S4, Supporting Information). The whole system was printed as a single hydrogel piece around an elastomer pillar that served as the shaft for the constrained rotation (Figure 2d). We tuned the design of the arm by considering the following trade‐off; thin arms lead to parasitic oscillations during operation while thick arms generate large viscous drag. The angular velocity of the µrotor was proportional to the square of the input voltage or applied acoustic pressure (Figure S7, Supporting Information). The angular velocity was as high as 1200 rpm at 5.1 kHz and *V*_PP_ = 55 V, which is orders of magnitude higher than velocities recorded with magnetic,^[^ ^21^ ^]^ acoustic,^[^ ^38^ ^]^ or optical µrotors.^[^ ^25^ ^]^ The efficient transduction of acoustic energy into mechanical work was due to the relatively high forces generated by acoustic streaming and hydrodynamically favorable 3D design of the engine and the arms of the rotor. We performed experiments to demonstrate that rotation was primarily generated by the µthrusters and not due to bulk streaming in the channel or emergence of standing waves. Once released from the substrate and excited with acoustic waves, µthrusters moved around freely along the opposite direction of the sharp‐edged opening, verifying the thrust generation mechanism (Movie S5, Supporting Information). In addition, rotors without µthrusters did not respond to acoustic excitation at/around operational frequency from 3 to 6 kHz.

To study the dynamics of motion, we developed a one‐to‐one 3D model of the mechanism along with the µthrusters. The flexible body of the rotor has its own resonance modes and some of these modes facilitate the actuation of the µthrusters through vibration of the arms (Figure 2e). At higher frequencies, the arms showed much less movement while deformation was observed primarily at the tip of the µthrusters (Figure S8, Supporting Information). We calculated the thrust generated by each engine using the 3D model of the µrotor. All engines were identical, which allowed us to divide the computational domain into quadrants. An extra symmetry plane perpendicular to the rotor axis was formed to further reduce the size of the domain and 1/8th of the structure was simulated. A single rotating reference frame was created around the rotor and angular velocity was set as an input parameter. This method provides an approximation for the steady‐state flow field around the rotor in its reference frame without going through the complexity of modeling moving parts. The total pressure along with the viscous stresses acting on the structure was integrated to compute the total fluid force or drag for experimentally recorded angular velocities. Since the rotor was operated at steady state, it was possible to directly deduce thrust from drag (Figure 2f). According to the simulation results, each µthruster generates 9.15 nN at *V*_PP_ = 50 V (Figure 2f). These calculations give an order of magnitude estimate on the thrust generated upon acoustic excitation and guide future development of microscale turbomachinery.

One shortcoming of the presented prototype is the lack of directional control (i.e., the rotor rotates only in one direction). The response of the structures to acoustic waves depends on its architecture, thus, the distribution of forces can be controlled by engineering multiple µthrusters with varying wedge design. We engineered a µrotor that rotates in both clockwise (CW) and counterclockwise (CCW) by incorporating two types of µthruster that resonate at different frequencies (Figure 2g). They were arranged as couples to preserve the symmetry of the rotor and minimize off axis motion. We performed a numerical modal analysis of the machine for frequencies up to 200 kHz and determined two frequencies, 5.19 and 25.77 kHz, at which µthrusters were selectively excited (Figure 2h). In the experiments, these modes manifested at slightly different frequencies (6.7 and 22.7 kHz), which could be explained by fabrication imperfections (Movie S6, Supporting Information). The µrotor rotated 1.75 times faster at 6.7 kHz compared to the speed at 22.7 kHz for the same input power. Next, we explored the potential of sound waves in precise angular position control by modulating the input signal (Figure 2i and Movie S9, Supporting Information). To simplify the dynamics of machinery, we reduced the number of arms and fabricated a mechanism that can serve as a rotary positioner or steering wheel. This configuration resembles the thrust vectoring devices engineered for steering rockets and satellites using reaction engines. Angular displacement was controlled in steps using a technique called pulse‐width modulation (Figure 2j). Each pulse consisted of several cycles of a sine wave that were generated at the resonance frequency (*f*_s_ = 1/*T*_s_) of the µthrusters (4.6 and 23.8 kHz). We fixed the period of the pulse (*T*_P_) to *T*_P_ = 1 s and amplitude to *V*_PP_ = 40 V, and recorded the angular velocity of the positioner for varying pulse width (*w*) or duty cycle (Figure 2k). The same pulse generated slightly different angular displacement during the rotation. This variation can be attributed to the nonlinear propagation of acoustic waves in our experimental setup. Closed‐loop feedback control of input voltage may deliver precise and repeatable motion. For the same pulse width, we recorded slightly different angular displacement in CW and CCW directions. To compensate for this variation, we selectively tuned the pulse width to 1000 *T*_s_ and 1200 *T*_s_ for 4.6 and 23.8 kHz, respectively. This adjustment led to the same average angular velocity in both directions of motion (Figure 2l). This demonstration shows that a separate calibration curve is required for each degree of freedom.

After building a repertoire of micromachines, we explored the potential for robotic micromanipulation of synthetic and biological samples. As a proof of concept, we engineered devices for the controlled collection of microparticles and mammalian cells. With these first demonstrations, we envision to discover design rules for the development of remotely powered and programmable microdevices that can perform in vivo diagnostic or therapeutic operations. We upgraded the µjet engine by incorporating a collection chamber and a sieve into the original design as shown in **Figure** **3**a. The integrated device can circulate a suspension through the chamber to selectively collect target samples (Figure 3b). The sieve, together with the slits printed around the chamber, was designed to filter particles within the desired size range (Figure S9, Supporting Information). We first tested the functionality of the machine using 10 µm polystyrene microparticles. Figure 3c shows snapshots from the collection procedure where the chamber was gradually filled with particles (see Movie S8, Supporting Information). The granular nature of the suspension allowed the fluid to flow through the device until the chamber was completely jammed and there was almost no space left between the particles. The successful transport of particles from the inlet to the chamber depended on the dimension of the opening of the wedge, the acoustic power, and the size of the particles.

**Figure 3:**
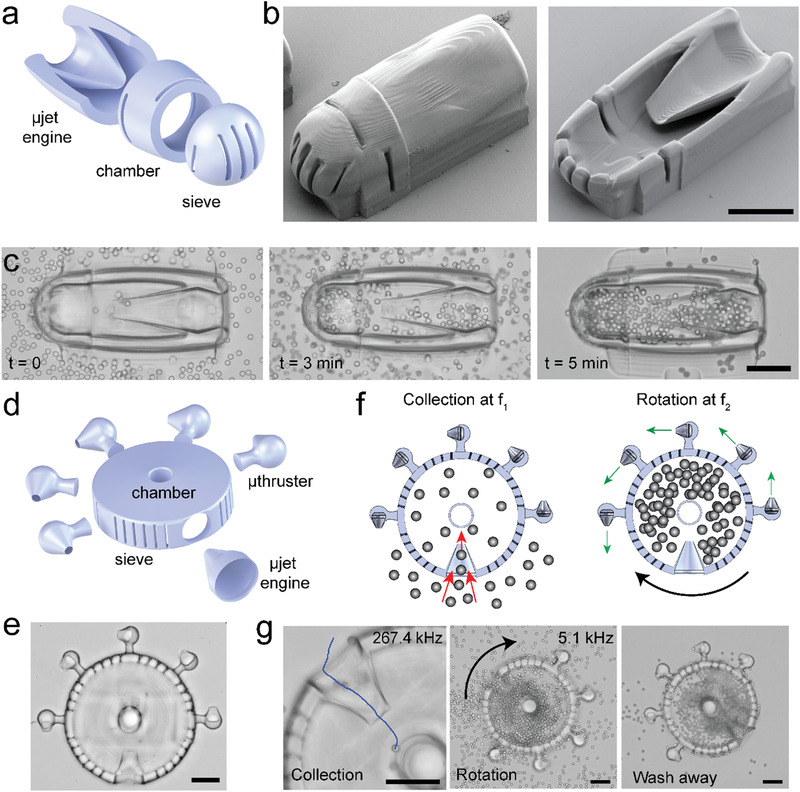
Soft robotic micromanipulation with compound machinery. a) Schematic illustration and b) electron microscopy images of a collection device comprised of a µjet engine, a chamber with slits to facilitate fluid flow, and a sieve for size‐selective particle and cell encapsulation. Images in (b) show fully (left) and partially (right) printed devices. c) Time‐lapse microscopy images from a particle collection experiment showing: i) the initial condition where the microfluidic chamber was filled with particles and the chamber of the device is empty (*t* = 0), ii) an intermediary state where the chamber was gradually filled with particles due to the acoustic powering of the µjet engine at 117 kHz, and iii) the final state that showed the completely filled chamber after washing away the free particles. d) Schematic illustration and e) bright‐field image of a motorized collection device. Several µthrusters are located around the chamber for effective control over angular motion. f) Illustration summarizing the working principles of the device. Addressable actuation of µjet engine and µthrusters enable multiple degrees of freedom control over the operation. g) Image sequence showing particle collection at 267.4 kHz, rotation of the filled device at 5.1 kHz, and the final state of the device after cleaning of the free particles. Scale bars: 75 µm.

Next, we tested the functionality of our device under physiologically relevant environmental conditions. We filled the microfluidic chamber with human embryonic kidney cells that were suspended in culture medium (Movie S9, Supporting Information). Movie S10 in the Supporting Information shows examples of individual cells going through the constriction while being transported by the device. Notably, wedge opening diameter smaller than 15 µm did not allow collection of cells even though cells should be able to fit into this constriction. We observed that few cells that could pass in the beginning of the experiment got immediately trapped in the steady streaming around the wedge and clogged the channel. On the other hand, when we increased the diameter of the opening above 40 µm, the pumping efficiency went down significantly because the effectiveness of the jet was gradually lost. Another interesting feature of acoustofluidic manipulation was that nearby cells attracted each other due to secondary acoustic radiation force or secondary Bjerknes force,^[^ ^42^, ^43^, ^44^ ^]^ which facilitated mass transport in our experiments.

As a final demonstration, we combined local pumping with rotary motion to construct a multibody particle collection device with angular position control (Figure 3d). The engines in this modular device were chosen from the repertoire of machines shown in the previous sections. The compound machine was designed in a way that the µjet engine and µthrusters were excited at two different frequencies (Figure 3e). Upon acoustic excitation at 267.4 kHz, 10 µm polystyrene microparticles were started to be transported into the main chamber (Movie S11, Supporting Information). At this relatively high frequency, we observed no streaming around the µthrusters and the device stayed stationary. After filling the chamber, we activated the µthrusters at 5.1 kHz, which led to the continuous rotation of the device as expected with no detectable pumping of particles. The device retained the entrapped particles inside the chamber during the washing away of the free particles (Figure 3f). This multifunctional modular device allowed on‐demand collection and manipulation of particles at specified locations inside the microfluidic channel. As a future extension, the device can be fully mobilized to enable both translational and rotational motion synchronized with particle collection. For precisely controlling the direction of motion, µthrusters with multiple resonance frequencies must be operated with appropriate control signals, as shown in Figure 2e,h.

Unlike alternatives based on electromagnetic, chemical or optical actuation, our powering scheme does not require the use of any specific material or medium. In the presented actuation paradigm, small amplitude structural oscillations of machine components at resonance generated large scale motion and mass transport. This strategy provides effective and efficient means for harnessing power from traveling acoustic waves, an important feature for in vivo applications. To further evaluate clinical potential, we verified the functionality of all the prototypes using medical grade immersion and contact ultrasound transducers (Figure S10, Supporting Information). We chose hydrogels as the material for manufacturing because they have tunable Young's modulus (Pa to MPa) that is comparable to biological tissues. Furthermore, there are hydrogel formulations that are biocompatible, biodegradable, and amenable to functionalization for biochemical stimulation of target cells. These are other key properties for the intended in vivo applications. Notably, hydrogels can possess physical intelligence through stimuli‐responsive behavior.^[^ ^14^ ^]^ By tailoring the material composition of the resin, we can build autonomous microrobots that can reconfigure their shapes in response to changes in their microenvironment. This morphological transformation can be directly coupled to our acoustic powering paradigm based on sharp‐edged structures, closing the loop for sensing‐computation‐actuation cycle, which is the basis of adaptive behavior and autonomy. Taken together, with the introduction of a proper injection or implantation technique, the presented machinery has the potential to become accessible for clinical applications.

There is no formal methodology for the design of monolithic compliant machines with multiple vibrating engines. With the incorporation of every new engine, the resonance modes of all the existing engines and the corresponding streaming profiles change. The integrated response of the machine articulates the importance of computational analysis. We have developed a modeling framework that can be used as a design tool for the development of more complex microrobots. Considering the small amplitude deformation, it is unlikely that fluid has a significant effect on the bending of the structures. As a result, our modeling assumption on one way coupling from the solid to the fluid seems reasonable. However, in our simulations, we completely ignored the propagation of sound waves in the media. These effects are expected to be more dominant inside biological tissues. We observed that with increasing frequency the velocity of the bulk streaming that appears around the casing reduces significantly. Thus, intuitively, operating devices at high frequencies will increase the precision of manipulation and enhance the validity of computational design efforts. In our future work, we will extend the model to incorporate the direct interactions between acoustic waves and surrounding media. Recent work with macroscale mechanical metamaterials has shown that locally varying the shape or stiffness of the building blocks and exploiting interactions of transition fronts with topological defects lead to predictable and programmable nonlinear motion.^[^ ^45^, ^46^, ^47^ ^]^ The development of acoustically powered multibody systems may lead to creation of mechanical logic and reconfigurable architecture toward the realization of more efficient and autonomous microrobotic devices.

## Experimental Section

##### Fabrication of Soft Robotic Microsystems

Hydrogel structures were fabricated using a 3D laser nanoprinting machine (Nanoscribe GmbH). A 63x oil immersion objective (63×/1.4 Oil DIC M27, Zeiss) was used for printing structures inside closed microfluidic channels from a solution of PEGDA (*M*_n_ = 250, Sigma) and pentaerythritol triacrylate with 3:1 ratio (v/v). A photosensitizer (4,4′‐bis(diethylamino)benzophenone) and photoinitiator (Irgacure 369, Sigma) were added to at a final concentration of 1% and 5% w/v, respectively. 3D models of the structures were sketched using a computer‐aided design (CAD) software (Solidworks 2018, Dessault Systèmes) and further processed via Describe (Nanoscribe GmbH) before importing them for printing. 3D models were sliced 0.25 µm vertically and 0.05 µm horizontally with a hatching angle of 45°. A scan speed of 0.3 m s^−1^ and laser power of 45 mW were set for all printing jobs. The unexposed hydrogel solution was gently washed away with ethanol. Hydrogel structures with large surface area adhere strongly to untreated glass substrates. To avoid adhesion issues, mobile structures were printed in 3D forms that minimized contact with the substrate. In addition, printing parameters were chosen to adjust the first print layer slightly above the glass slide, which greatly reduced adhesion to the substrate. As a result, gentle agitation applied by the flow seamlessly released the structures. Devices were kept in ethanol for long‐term storage.

##### Fabrication of Microfluidic Devices

Microchannels were fabricated using replica molding. Briefly, 5 µm positive photoresist (AZ9260, MicroChem) was spin coated on a 4‐in. silicon wafer using automatic resist processing cluster (EVG150, EVG Group). Photolithography was performed using a mask aligner (MJB4, Süss) and the mold was prepared using reactive ion etching (AMS 200, Alcatel Adixen). The topography was mapped using a mechanical profilometer (Dektak XT, Bruker). The mold was silanized (trichloro(1H,1H,2H,2H‐perfluorooctyl) silane, Sigma) under vacuum for 6 h. Poly(dimethylsiloxane) was prepared as a mixture (10:1, w/w) of elastomer and curing agent (Sylgard 184, Dow Corning) and poured on the mold in a petri dish. After degassing of the mixture in a vacuum chamber, the elastomer was cured at 65 °C for 4 h. After the inlets and outlets were formed using a 0.5 mm biopsy punch (Elveflow), the device was bonded to a glass coverslip right after functionalizing the surfaces of both substrates with oxygen plasma (PDC‐32G, Harrick Plasma).

##### Experimental Platform

A piezo transducer (SMMOD15F120, Steminc INC.) was glued right next to the microfludic chamber using a 5 min epoxy (G14250, Devcon) as shown in Figure S11 in the Supporting Information. Solutions and suspensions were injected into the microfluidic device using a computer controlled syringe pump (neMESYS 290N, Cetoni). Before each experiment, channels were filled with ethanol to reduce the surface tension and avoid air bubble formation. The transducer was controlled by a function generator (AFG‐2225, Gw Instek) connected to a high voltage amplifier (HVA200, ThorLabs). Soft robotic microsystems were also excited using water immersion (GS200‐D19‐P50, The Ultran Group) and contact transducers (GC100‐D19, The Ultran Group) to demonstrate acoustic wave transmission in different configurations.

##### Microscopy and Imaging

Acquisition of images and videos was performed using a high speed camera (VEO640L, Phantom) connected to an inverted microscope (Ti2, Nikon) with a 20X objective (Nikon). Videos were captured using the camera‘s commercially available software (phantom camera control, Phantom) and analyzed using Fiji (National Institute of Health). Data were plotted using Prism 8 (GrapPad Software Inc.). Streamlines were visualized using 1 µm fluorescent polystyrene particles (Cat # 17154‐10, Polysciences). High‐magnification images were taken using a scanning electron microscope (LEO 1550, Zeiss). Prior to imaging, devices were fixed using hexamethyldisilazane (Sigma) to preserve their structure and coated with a thin conductive layer of gold (10 nm) using sputtering (DP650, Alliance‐Concept).

##### Numerical Simulation

CAD models generated in Solidworks and CatiaV5 were imported to COMSOL Multiphysics 5.4 for mesh generation and computational analysis. 2D simulations were performed with triangular elements while 3D simulations were performed with tetrahedral elements to ease the meshing process. An adaptive mesh scheme was followed (Figure S12, Supporting Information). For example, 90 000 tetrahedral elements were used for the mesh of engines (minimum quality of 0.3 and average quality of 0.9) that are on the order of 100 µm in size with a surrounding fluid domain that was at least four times larger than the volume of the structure. The area around the tip was refined with 0.05 µm mesh elements. The selected time step was 0.02 µs for the simulations. These values were chosen in order to ensure that the mesh was converged and enough data points were saved to accurately capture the mechanics of the simulated models. The eigenfrequency analysis module was used to extract the eigenmodes and eigenfrequencies of 3D structures. The values of the density of the hydrogel, Young's modulus of the hydrogel, and the viscosity of the fluid are given by *ρ* = 1000 kg m^−3^, *E* = 0.5 MPa, and *μ* = 0.001 Pa s, respectively. The presence of the surrounding liquid was incorporated as added mass on the structure. The normalized eigenmodes were used as inputs for the CFD simulation. A coefficient was tuned manually to define the amplitude of the oscillations. The resulting eigenmode shape was used as a moving boundary in the CFD simulation and it was assumed that the fluid‐structure interactions were only in one way (i.e., from solid to fluid). The Laminar module combined with the moving mesh module (Arbitrary Lagrangian‐Eulerian) was used to obtain the flow patterns around the oscillating structures. The viscous stresses and the total pressure were integrated to extract the total stresses on the structure.

##### Cell Culture

HEK 293T/17 (American Type Culture Collection) were cultured in Dulbecco's modified Eagle medium GlutaMAX (LifeTechnologies, Carlsbad, CA, USA) supplemented with 10% fetal bovine serum (LifeTechnologies) and 1% penicillin‐streptomycin (LifeTechnologies). Cells were passaged every 2–3 d using trypsin 0.25% (ethylenediaminetetraacetic acid, LifeTechnologies) and not kept longer than 20 passages. All experiments were done with cells that tested negative for mycoplasma.

##### Statistical Analysis

Results are presented as mean ± standard deviation. Statistical analysis was performed using Prism 8 (Graphpad). Data were processed coming from at least three different trials per device and the number of devices tested (*n*) is at least 4 (*n* > 3) for each condition.

## Conflict of Interest

The authors declare no conflict of interest.

## Supporting information

Supporting Information

Supplemental Movie 1

Supplemental Movie 2

Supplemental Movie 3

Supplemental Movie 4

Supplemental Movie 5

Supplemental Movie 6

Supplemental Movie 7

Supplemental Movie 8

Supplemental Movie 9

Supplemental Movie 10

Supplemental Movie 11
