# Supplementary material for: Addressable Acoustic Actuation of 3D Printed Soft Robotic Microsystems
Source: Adv Sci (Weinh). 2020 Sep 21;7(20):2001120. doi: 10.1002/advs.202001120 (PMC7578873; doi:10.1002/advs.202001120)
Supplement: Supplementary file 1 — Supporting Information [file ADVS-7-2001120-s001.pdf]

© 2020 Wiley-VCH GmbH

ADVANCED SCIENCE
Open Access

Supporting Information

for *Adv. Sci.,* DOI: 10.1002/advs.202001120

Addressable Acoustic Actuation of 3D Printed Soft Robotic Microsystems

*Murat Kaynak, Pietro Dirix, and Mahmut Selman Sakar**

Supporting Information

Addressable Acoustic Actuation of 3D Printed Soft Robotic Microsystems

*Murat Kaynak, Pietro Dirix, and Mahmut Selman Sakar**

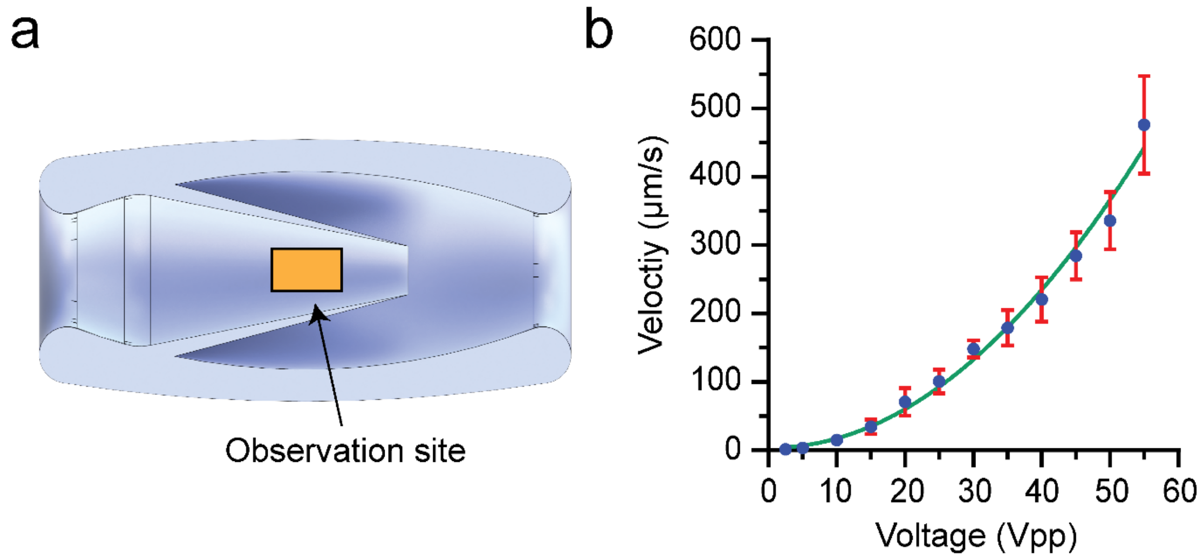

Figure S1. The fluid flows from inlet to outlet along the pump. a) The observation site, where the data is collected, is located between ~30 to 50 µm behind the wedge tip. b) Flow velocity along the longitudinal axis shows a quadratic relationship with the input voltage. Error bars represents the SD of the mean.

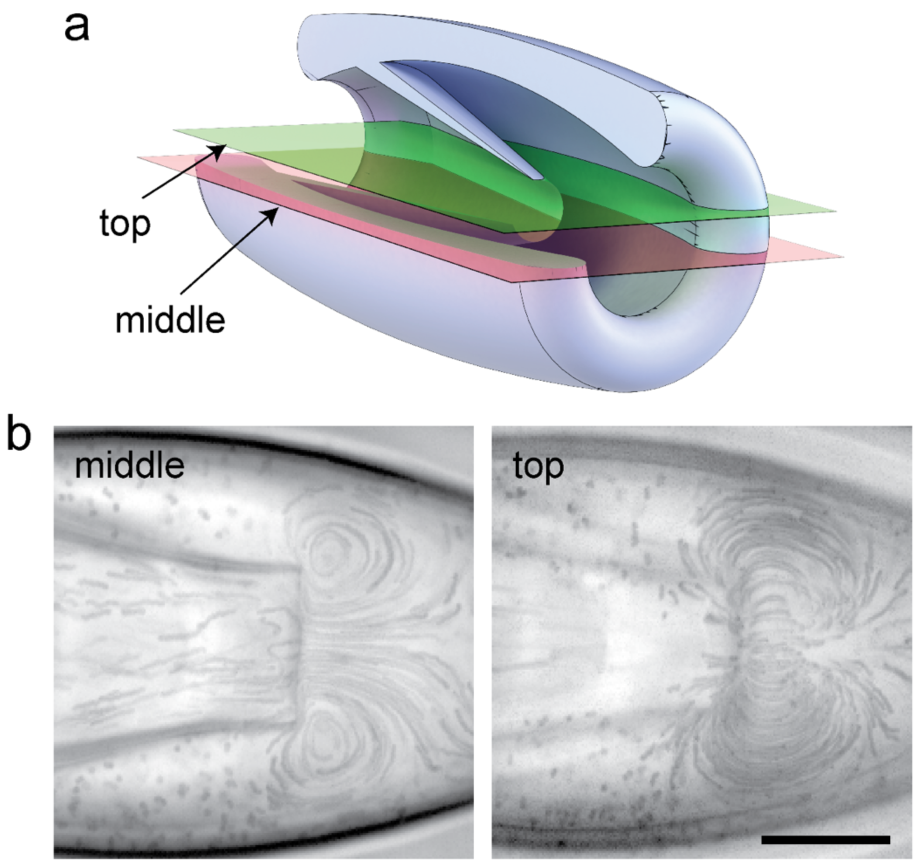

Figure S2. The streaming forms a vertex ring around the tip of the wedge. a) Representative schematic of the layers in the middle and at the top of the wedge. b) The jet in the middle layer results in pumping while couter-rotating vortices on the both sides are in steady state. c) The streaming at the top layer of wedge tip leads to continuous vortices. Scale bar is 25 µm in b)

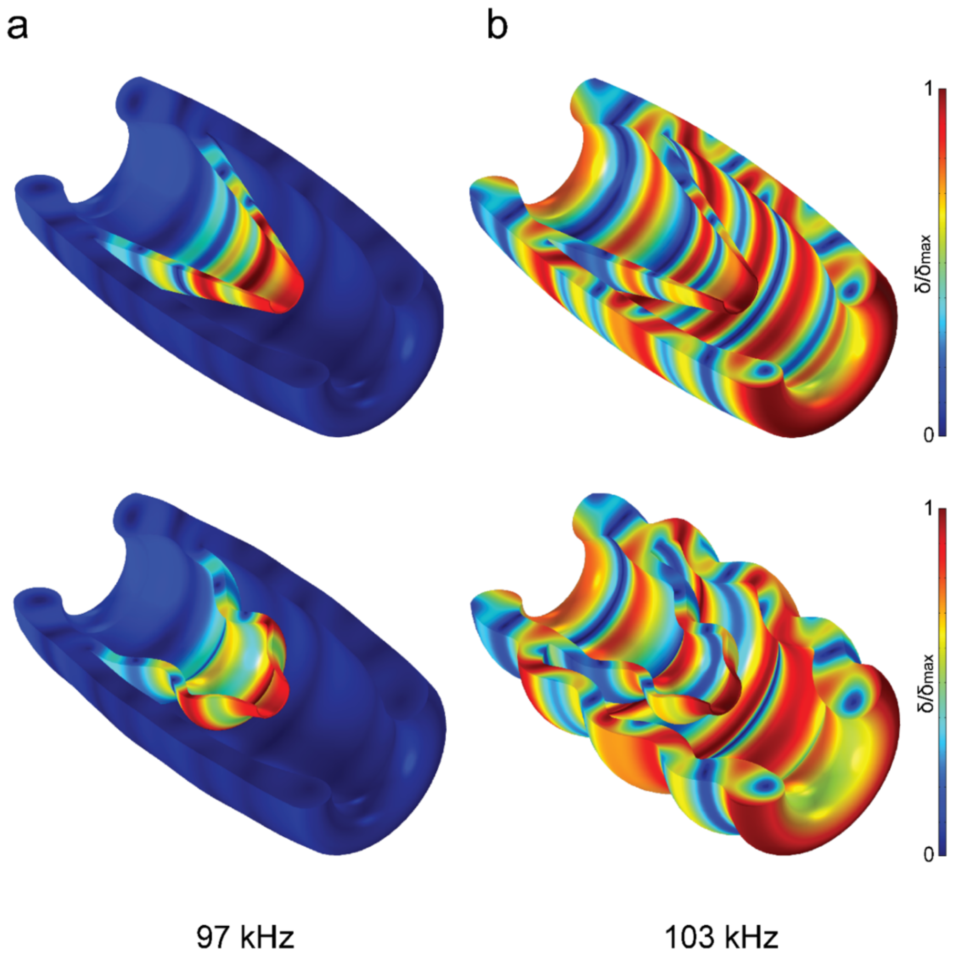

Figure S3. Representative eigenmodes of the pump. a) Displacement localized at wedge leads to fluid flow pumping. b) Dispersed displacement throughout the pump results in unwanted streaming inside and outside of the pump with limited flow pumping. Top panel shows color coding only while bottom panel shows color coding with exaggerated deformation.

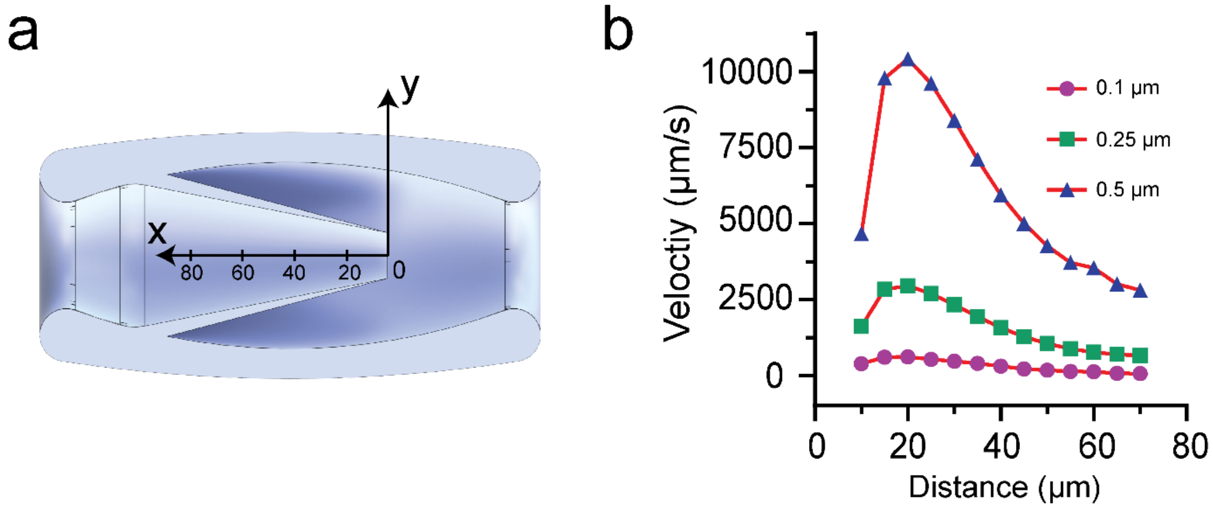

Figure S4. Computational approximization of the tip displacement. a) A schematics showing the axis where the data collected in the middle. b) The flow velocity increases with tip displacement. Additionally, the flow velocity in the x-direction rises up until 20 μm away from the wedge tip. Then, the flow is under the effect of streaming resulting in changes in the flow direction.

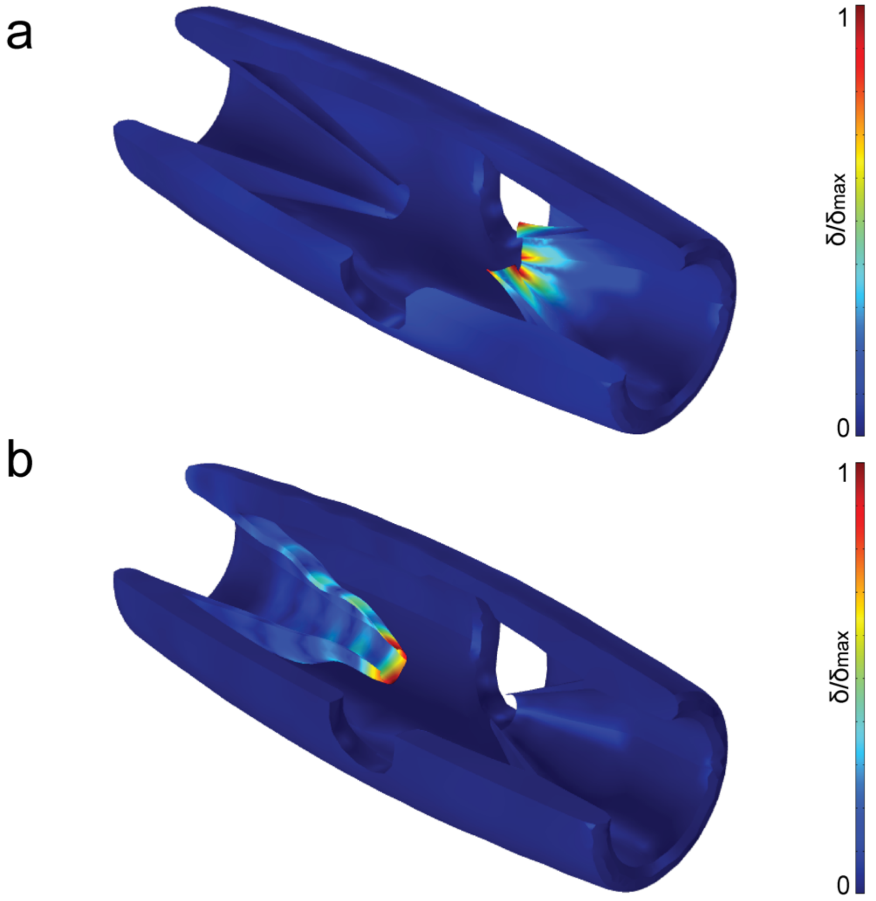

Figure S5. Deformation profile of the bidirectional pump at (a) 42.3 kHz (corresponding to Figure 1h, top) and (b) 119.2 kHz (corresponding to Figure 1h, bottom). The deformations are exaggerated for clarity.

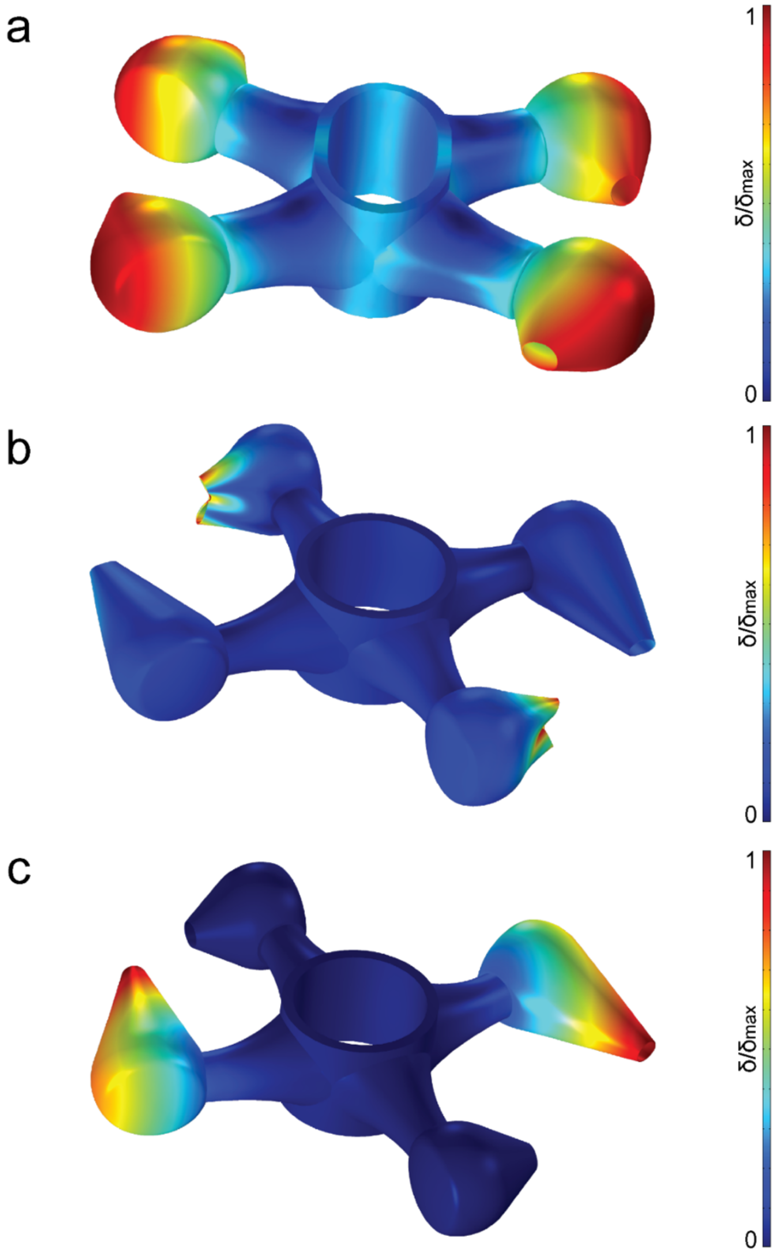

Figure S6. Deformation profile of the bidirectional *rotor* at *(*a) 3.75 kHz corresponding to Figure 2e), *(*b) 25.77 kHz (corresponding to Figure 2h, left) and *(*c) 5.19 kHz (corresponding to Figure 2h, right). The deformations are exaggerated for clarity.

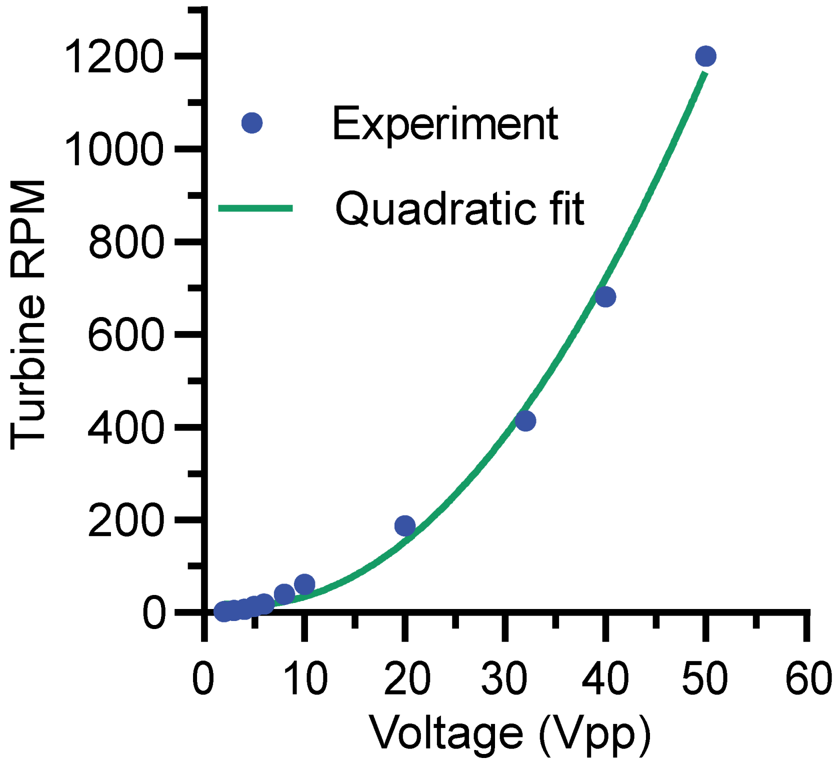

Figure S7. The angular velocity of the μrotor changes quadratically with the input voltage applied to the transducer.

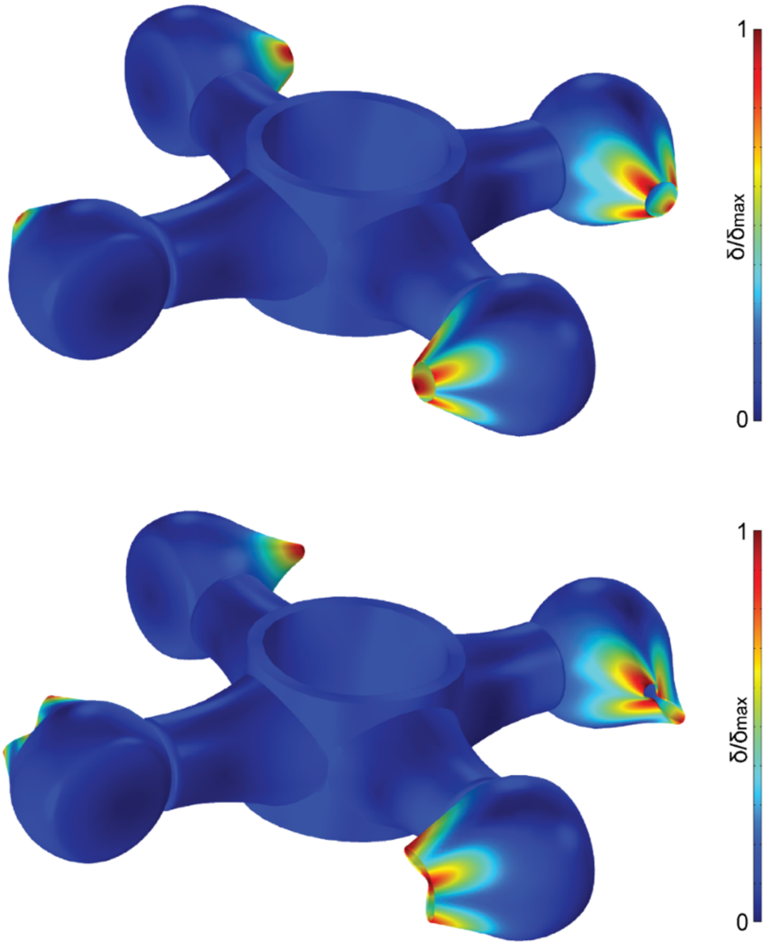

43 kHz

Figure S8. A representative eigenmode of the 4-arm µrotor along driven by µthrusters at a higher resonance frequency. The deflection is localized at the tip of µthrusters resulting in less rotational speed, yet smooth rotation in a rotation compared to lower frequency resonance mode. Top panel shows color coding only while bottom panel shows color coding with exaggerated deformation.

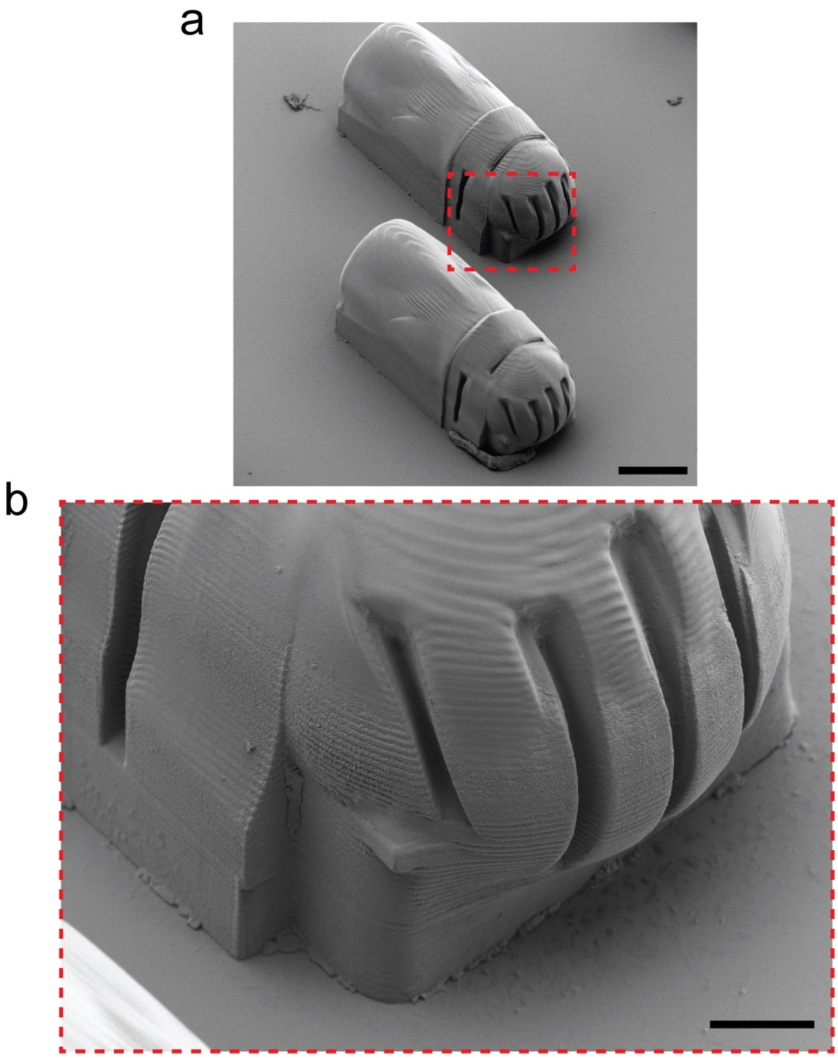

Figure S9. SEM images of the μjet engine integrated with a collection chamber and a sieve to collect particles and cells. a) Two identical integrated devices are fabricated next to each other. b) Close-view image of the sieve. Scale bars: a) 75 um, b) 20 um.

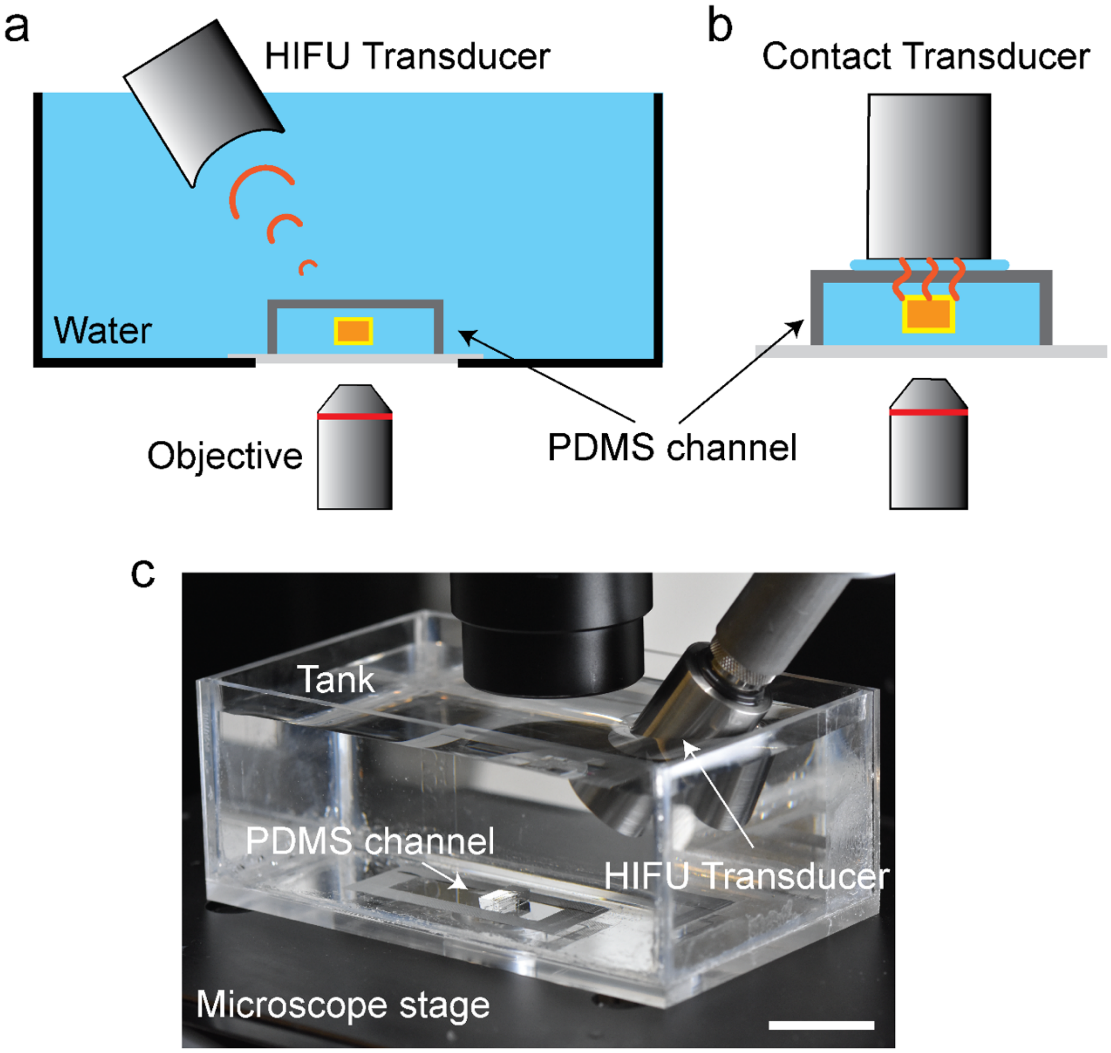

Figure S10. Clinically relevant actuation. a) Water immersion high intensity focused ultrasound (HIFU) transducer is positioned above the µchannel where the µthrusters and µjet engines are 3D-printed in situ. b) Contact ultrasound transducer located above the µchannel with water as a coupling agent in between. c) A picture showing the water-filled tank, HIFU and µchannel placed on a microscope stage. Scale bar is 4 cm.

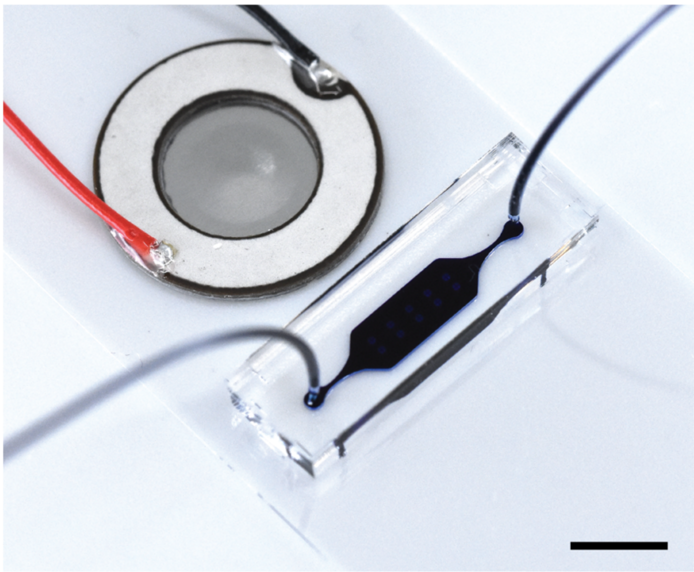

Figure S11. A picture of the experimental platform. μthrusters and μjet engines are fabricated inside an enclosed μchannel. Then, they are actuated via piezotransducer placed adjacent to the μchannel. Scale bar is 5 mm.

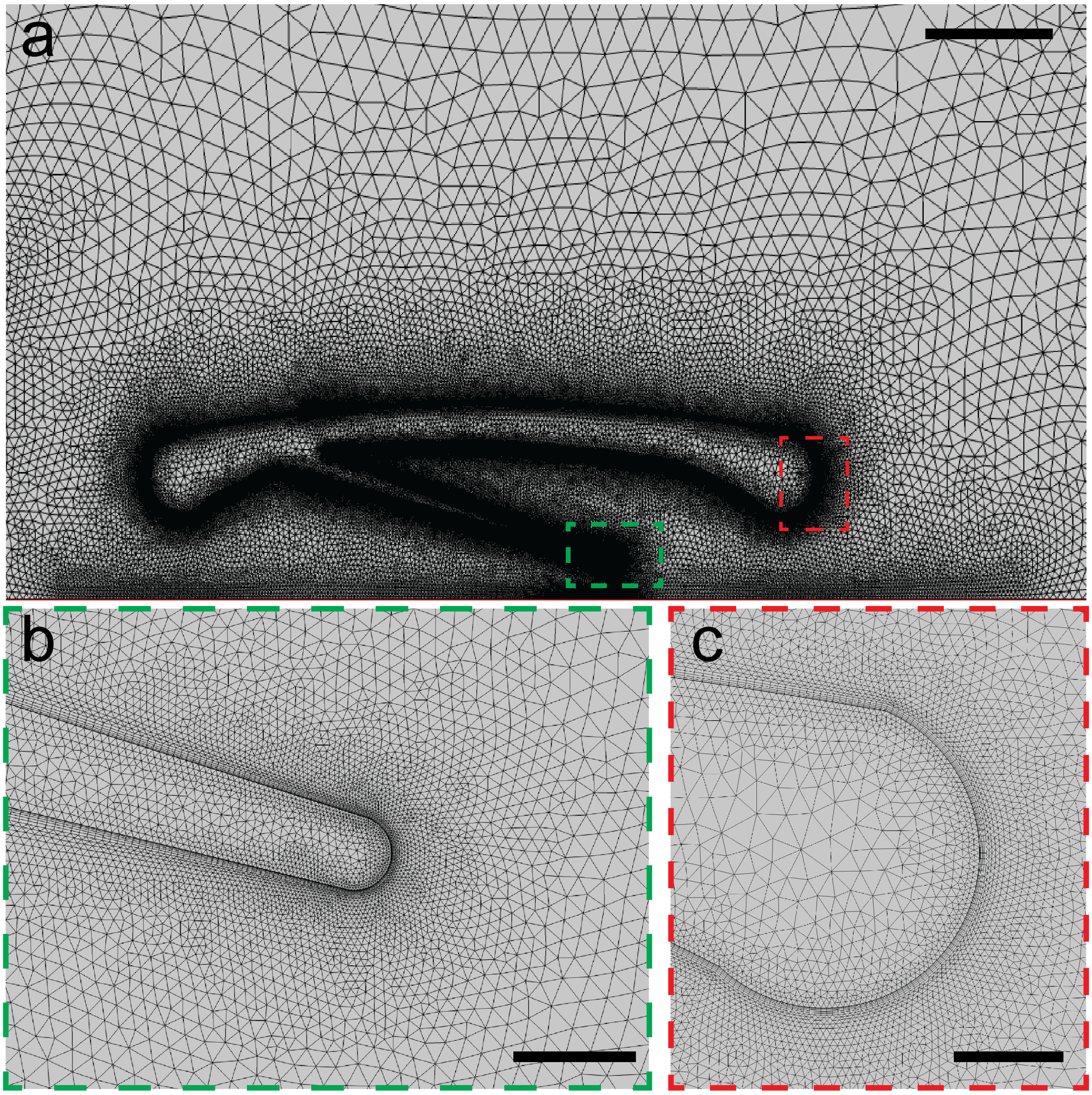

Figure S12. Adaptive mesh formation for numerical simulation. (a) A representative example showing the varying mesh size on and around a µjet engine. Magnified views of (b) the tip of the wedge and (c) the tip of the capsule shows mesh refinement. Scale bars: (a) 50 µm, (b) 2 µm, and (c) 10 µm.

Supplementary Movies

Movie S1. Microfluidic pumping of fluids using acoustically powered μjet engine

Movie S2. Particle image velocimetry showing the vortices and jet around the wedge

Movie S3. Bidirectional pumping at two different eigenfrequencies

Movie S4. Rotation of 3D printed μrotor driven by several μthrusters

Movie S5. The translational motion of an untethered μthruster

Movie S6. Bidirectional rotation at two different eigenfrequencies

Movie S7. Precise control of angular displacement using pulse width modulation

Movie 8. Bead collection using an integrated device with μjet engine, chamber, and sieve

Movie 9. Mammalian cell collection using the integrated device

Movie 10. Tracking single cells transported through the wedge

Movie 11. Collection and manipulation of particles at two different eigenfrequencies
